# Unveiling the Culprit: Candida-Induced Spondylodiscitis Following SARS-CoV-2 Infection

**DOI:** 10.7759/cureus.42079

**Published:** 2023-07-18

**Authors:** Mohammad O Khalil, Laith A Ayasa, Anas Odeh, Husain Alawadhi

**Affiliations:** 1 Faculty of Medicine, Al-Quds University, Jerusalem, PSE; 2 Internal Medicine, Al-Quds University, Jerusalem, PSE; 3 Department of Medicine, An-Najah National University, Nablus, PSE; 4 Critical Care, Sheikh Shakhbout Medical City, Abu Dhabi, ARE

**Keywords:** sars-cov-2 infection, fungal infections, candida albicans, spondylodiscitis, covid-19

## Abstract

We present a case of a 57-year-old male patient with a history of prolonged intensive care unit (ICU) stay for coronavirus disease 2019 (COVID-19) who developed fungal spondylodiscitis, a rare complication. The patient initially presented complaining of respiratory symptoms and was subsequently treated with tocilizumab, remdesivir, enoxaparin, and dexamethasone. Following ICU discharge, he experienced recurrent infections, including extended-spectrum beta-lactamase *Klebsiella* urinary tract infection. Two months later, he developed back pain; magnetic resonance imaging (MRI) revealed inflammatory spondylodiscitis. Despite empirical antibiotic therapy, his condition did not improve, and a bone biopsy confirmed *Candida albicans* infection. Antifungal treatment with fluconazole and anidulafungin resulted in a significant clinical improvement. The patient achieved complete recovery after six months of therapy. This case highlights the rare occurrence of fungal spondylodiscitis in COVID-19 patients with a history of ICU stay and emphasizes the importance of early recognition and appropriate management to mitigate potential complications.

## Introduction

The coronavirus disease 2019 (COVID-19) pandemic has had a devastating global impact over the past few years. The healthcare sector was faced with a steep rise in opportunistic infections evident in severe acute respiratory syndrome coronavirus 2 (SARS-CoV-2) patients’ course of management. Consequently and despite their scarce incidence, it is paramount to document and recognize some of these complications, given their non-negligible mortality and specific management requirements.

Spinal infections are considered a rare entity because they constitute only 1% of infections involving the bones [1]. Most of these infections have a pyogenic or tuberculosis origin. Fungal infections, however, are considered to have a very rare occurrence and could complicate lengthy intensive care unit (ICU) stays and immunosuppressive status [2].

In this report, we present a rare, unfamiliar case of fungal spondylodiscitis affecting a patient infected with SARS-CoV-2 with a history of a prolonged ICU stay. We comprehensively document his clinical course and review the available relevant literature.

## Case presentation

A 57-year-old male patient of Pakistani origin with no comorbidities presented to the emergency department (ED) with a three-day history of fever, cough, and loss of taste and smell. Following evaluation, he was diagnosed with acute respiratory infection due to coronavirus disease (COVID-19). A chest X-ray was performed, revealing bilateral lower-zone airspace opacification. Despite receiving supportive care, the patient's condition deteriorated, evident by a subsequent follow-up chest X-ray showing significant progression of opacification, now extending to the mid and lower zones. As a result, the patient was admitted to the hospital and initiated on intravenous (IV) tocilizumab (8 mg/kg, infusion over 60 minutes, single dose), IV remdesivir (100 mg, daily), subcutaneous enoxaparin (40 mg, every 12 hours), and IV dexamethasone (6 mg, daily).

**Figure 1:**
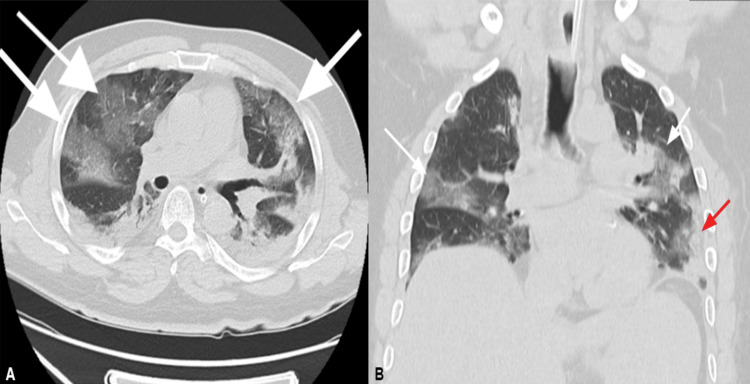
Chest CT scan showing bilateral lung consolidations (A) Transverse section chest CT scan showing bilateral patches with a ground glass appearance and consolidation with a crazy paving pattern in both lungs (white arrows). (B) Coronal section chest CT scan showing bilateral patches with a ground glass appearance and scattered fibrotic bands (white arrows) with thickened interlobular septa mainly at the periphery of the lungs (red arrow).

Subsequently, the patient experienced a severe decline in oxygen saturation, reaching 80%, necessitating his transfer to the intensive care unit (ICU) for closer monitoring and mechanical ventilation. After intubation, the patient was placed in a prone position to improve oxygenation by enhancing ventilation-perfusion matching [3]. Furthermore, the patient had a central line placed for invasive monitoring and administration of inotropes. A computed tomography (CT) scan was performed, revealing bilateral lung involvement characterized by a ground glass appearance and consolidation, displaying a distinctive crazy-paving pattern in both lungs (Figure 1A, B). Furthermore, the patient developed bacterial pneumonia, which was confirmed through sputum culture, identifying methicillin-sensitive *Staphylococcus aureus* (MSSA). To address this secondary infection, the patient received IV injection of ceftriaxone (2 g/day). After one week, a marked clinical improvement was observed, leading to the discontinuation of mechanical ventilation. A subsequent chest X-ray demonstrated significant improvement, characterized by decreased opacification. Following a total of 16 consecutive days of comprehensive management, the patient's condition improved, prompting his discharge from the ICU.

One week after his ICU discharge, the patient presented to the hospital again, complaining of fever, chills, increased urinary frequency, and pain during urination. A urine culture revealed an extended-spectrum beta-lactamase (ESBL) *Klebsiella* infection. Consequently, he was readmitted to the hospital and treated with IV ertapenem (1 g/day). After a week, urinalysis and urine cultures were normal, and the patient was discharged on oral fosfomycin (3 g, once). 

**Figure 2:**
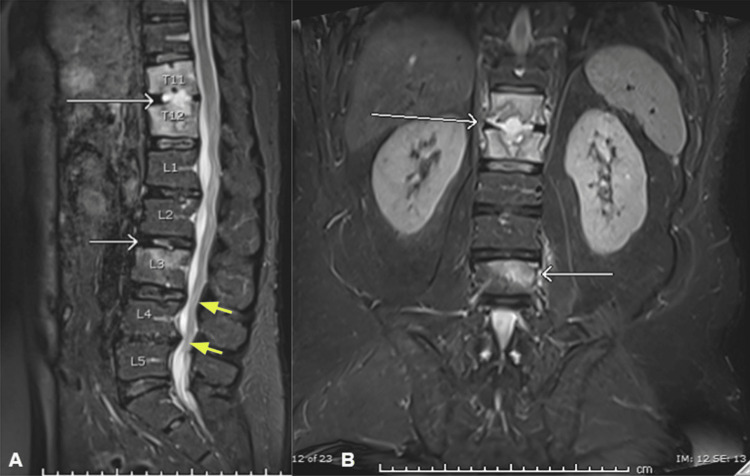
MRI at presentation showing inflammatory and degenerative changes (A) Sagittal MRI showing inflammatory process at T11-T12 and L2-3 disc levels (white arrows). Note the degenerative changes occurring in the lumbar spine, indicative of a spondylodegenerative condition (yellow arrows). (B) Coronal MRI showing the inflammatory process occurring in T11-T12 and L2-L3 disc levels (white arrows).

Three months later, the patient presented to the ED complaining of worsening mid to low back pain that had persisted for two months. A magnetic resonance imaging (MRI) was performed (Figure 2A, B), revealing an apparent inflammatory process observed at the T11-T12 disc level, with a lesser degree at L2-3; degenerative changes occurring in the lumbar spine, indicative of a spondylodegenerative condition; a diffuse disc bulge at L4-5, accompanied by hypertrophy of the ligamentum flavum and facet arthropathy, leading to central canal stenosis; and a minor disc bulge detected at L5-S1.

These findings were consistent with inflammatory spondylodiscitis. Consequently, the patient was admitted and started on IV ceftriaxone (1 g/day) and IV linezolid (600 mg every 12 hours) while monitoring inflammatory markers. The interferon-gamma release assay (IGRA) for tuberculosis yielded a negative result.

**Figure 3:**
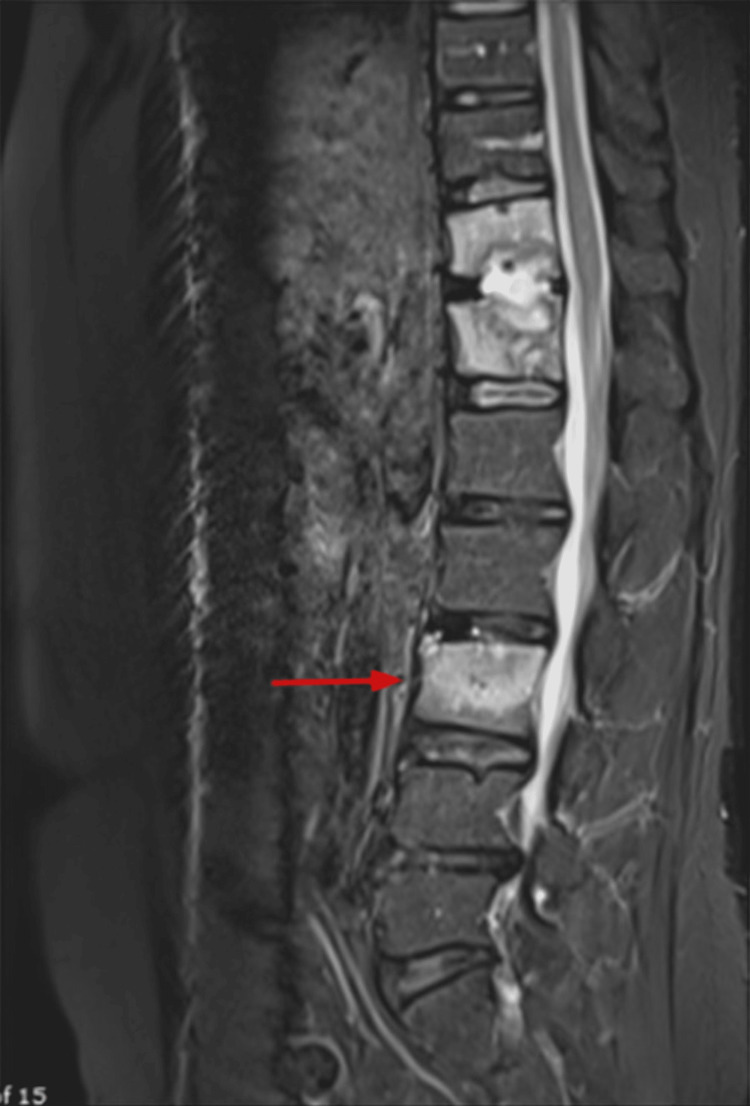
Sagittal MRI after two weeks of empiric antibiotic therapy Compared to Figures 2A and 2B, there is an increased bone marrow edema (red arrow) with no other significant changes.

Although the patient underwent a one-week trial of empiric therapy, the C-reactive protein (CRP) level was measured at 56 mg/L, and the erythrocyte sedimentation rate (ESR) was 95 mm/hr. Therefore, ceftriaxone was replaced with IV ertapenem (1 g/day), known for its superior bone penetration. Despite this change in antibiotics, there was no improvement observed as the plasma inflammatory markers remained elevated. Due to the severity of symptoms, a follow-up MRI was scheduled after two weeks to exclude abscess formation, revealing no significant changes (Figure 3). Initially, a CT-guided biopsy was recommended, but due to challenges with the radiology department, an open biopsy was done instead. The culture of the bone biopsy grew *Candida albicans*, indicating that his inflammatory spondylodiscitis was due to a fungal causative organism. Therefore, both ertapenem and linezolid were stopped, and instead, he was started on IV fluconazole (400 mg/day). After six days, the patient showed clinical improvement and was discharged on oral fluconazole for two months. However, despite being compliant with the treatment, the patient presented again with the similar symptoms and was readmitted. We inserted a peripherally inserted central catheter (PICC) line and started him on IV anidulafungin (200 mg loading dose, then 100 mg/day).

The patient was monitored with weekly CRP and ESR tests, as well as a beta-d-glucan test. Following the initiation of anidulafungin treatment and within one week, the clinical status of the patient significantly improved, as evidenced by the decrease in CRP from 59 (mg/L) to 26 (mg/L). The initial beta-d-glucan levels were 274 pg/mL, which decreased to 103 pg/mL after starting antifungal therapy, further confirming the fungal etiology. 

**Figure 4:**
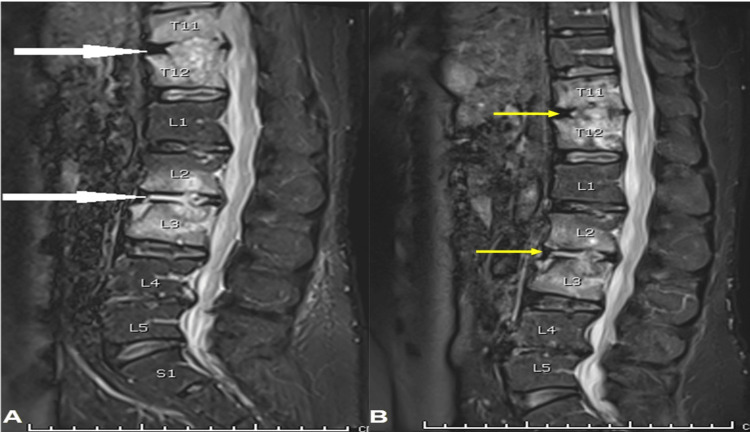
Sagittal MRI showing the inflammatory process at presentation and after four weeks (A) Sagittal MRI at presentation showing the large inflammatory process at the T11-T12 and L2-3 disc levels (white arrows). (B) Sagittal MRI after four weeks; inflammatory collections within the discs T11/T12 and L2/L3 levels still seen but decreased in size (yellow arrows).

Following notable improvement in his clinical status, the patient was discharged and requested to attend monthly follow-up appointments. Furthermore, a repeat MRI was scheduled after four weeks (Figure 4A, B), which revealed a decrease in the inflammatory processes at the T11-T12 and L2-3 disc levels and an attenuation in the bone marrow in the adjacent vertebral bodies. Remarkably, the patient achieved complete recovery within six months.

## Discussion

Spondylodiscitis refers to the inflammation affecting the vertebral bodies and intervertebral disk space. This condition is characterized by a complex nature with various contributing factors, necessitating a multidisciplinary approach to determine the most suitable intervention. It is a relatively rare yet significant infection, accounting for approximately 2-5% of all cases of osteomyelitis [4]. The occurrence of spondylodiscitis demonstrates certain patterns, with higher rates observed among individuals younger than 20 years old and those between the ages of 50 and 70 years [5]. Spondylodiscitis commonly occurs due to the dissemination of infection through the bloodstream, direct introduction during spinal surgery, or contiguous spread from an infection in the surrounding soft tissues. Among the microorganisms associated with pyogenic vertebral osteomyelitis, *Staphylococcus aureus* is the predominant pathogen, followed by *Escherichia coli *[6]. The connection between spondylodiscitis and COVID-19 is not clear, but there are several theories. It is thought that spondylodiscitis could arise from spontaneous bacteremia, bacterial pneumonia that has spread, or other infections acquired in the hospital during COVID-19 [7].

Among patients receiving intensive care for SARS-CoV-2, studies have indicated an increased prevalence of invasive fungal infections [8]. These individuals are at a higher risk of developing conditions, such as pulmonary aspergillosis, oral or hematogenous candidiasis, and pneumocystis pneumonia. The viral infection itself is associated with invasive fungal disease due to its attachment to angiotensin-converting enzyme 2 receptors located on respiratory epithelial cells. This viral attachment can temporarily hinder ciliary motility, reducing muco-ciliary clearance and impairing innate immune function [9]. The situation is worsened by the diminished ability of immune cells to engulf fungal spores as a consequence of gene downregulation involved in the process of recognizing, opsonizing, and destroying the spores [10]. In addition, the impact of immune exhaustion is significant in cases of COVID-19 infection, as the immune response of individuals, following even moderate illness, has been observed to deviate from anticipated patterns when encountered with fungal presence [10]. This virally induced defective immunity together with corticosteroid therapy and other immunomodulatory drugs can predispose patients to life-threatening infections. 

Fungal spondylodiscitis, especially caused by *Candida*, is uncommon and accounts for less than 5% of all spondylodiscitis cases [11]. The number of *Candida* infections has increased recently due to a rise in the population of at-risk individuals who have weakened immune systems and are exposed to high levels of antibiotics, often as a result of a fungal infection. In our case, the use of intravascular devices, the administration of broad-spectrum antibiotics and immunomodulatory drugs, and the patient's compromised immune system as a result of the COVID-19 infection all likely had an impact on the patient's clinical condition.

Clinical, laboratory, and imaging criteria determine the diagnosis of spondylodiscitis. It is characterized by a non-specific and often treacherous clinical presentation. Although fever has a weak sensitivity to the spondylodiscitis, as does for most other slow progressive infections, its absence should not deter healthcare providers from considering spondylodiscitis. The most common physical examination finding is localized back pain, particularly in the lumbar region, which is reported in over 90% of patients [12]. Point tenderness and pain upon percussion of affected vertebral bodies are frequently observed. Spinal cord or nerve compression is another significant manifestation of spondylodiscitis, often resulting in radicular compression and associated weakness, paresthesias, or paralysis [13]. Neurological symptoms should raise suspicion of abscess formation in the epidural space and therefore highlight the importance of early recognition and appropriate management of spondylodiscitis to mitigate potential complications.

Elevated levels of CRP and an increased count of white blood cells are common indicators of infections, although their relevance in detecting spondylodiscitis is limited. Blood culture is the simplest, most affordable, and highly effective approach for confirming the cause, particularly if obtained prior to administering antibiotics. A tissue sample for culture and histopathology testing is a basic requirement in order to determine the diagnosis of fungus etiology [14]. The addition of the beta-d-glucan test can be valuable in confirming the diagnosis of fungal etiology and monitoring the response to treatment, as observed in our patient. Imaging of fungal spondylodiscitis does not appear to be specific and resembles either tuberculous or pyogenic discitis. X-ray images of the spinal segments may occasionally reveal bone dissolution and opacity in the surrounding soft tissue, suggesting the presence of a spinal abscess. MRI is a more sensitive and specific method of diagnosing spondylodiscitis, making it the preferred choice.

Simeone et al. proposed several imaging and clinical predictors to consider in differentiating fungal spondylodiscitis from *Staphylococcus aureus* spondylodiscitis [15]. Fungal infections often present with focal paravertebral soft tissue abnormalities and partial disc involvement, accompanied by persistent back pain lasting for 10 or more weeks. In addition, current antibiotic use for a week or more and current intravenous drug use can also be indicative of fungal discitis/osteomyelitis. On the other hand, *Staphylococcus aureus *infections exhibit diffuse paravertebral soft tissue abnormalities and diffuse disc involvement. The associated back pain tends to be of shorter duration, lasting for 10 or fewer weeks. Moreover, a history of invasive instrumentation within the past year can suggest *Staphylococcus aureus* discitis/osteomyelitis.

The primary treatment for fungal spondylodiscitis revolves around the administration of antifungal medications, both intravenously and orally, over a span of 6-12 months [16]. Surgical intervention becomes necessary when indicators, such as spinal instability, neurological compression, progressing infection, persistent pain, and deformity, are present [17]. While minimally invasive spine surgeries have shown success in treating pyogenic infections of the spine, particularly in patients who underwent a double approach or required abscess drainage, there is a dearth of information regarding their efficacy in managing fungal spondylodiscitis [18]. Previously, these surgeries were reserved for milder cases with accompanying bone destruction. However, current standards prioritize open surgery due to the imperative for aggressive debridement and stabilization. The key objectives of surgery encompass debridement, histological sample acquisition, decompression, and stabilization of the spine. This comprehensive approach is essential, as decompression alone can potentially lead to the development of kyphosis [19].

Moreno-Gómez et al. were the first to report a case of fungal spondylodiscitis occurring after COVID-19 infection in a 47-year-old man, who, in contrast to our patient, had a history of invasive candidemia during his hospitalization treated with fluconazole [11]. Despite receiving early antifungal treatment, the patient showed no clinical improvement and eventually underwent successful surgery via a thoracotomy approach.

## Conclusions

Spondylodiscitis is a complex and relatively rare infection that affects the vertebral bodies and intervertebral disk space, requiring a multidisciplinary approach for accurate diagnosis and effective management. The association between spondylodiscitis and COVID-19 remains uncertain, with several potential mechanisms suggested, including spontaneous bacteremia, spreading of bacterial pneumonia, or other infections acquired during hospitalization. Although *Staphylococcus aureus* and *Escherichia coli* are frequently associated with bacterial infections, immunocompromised patients can also experience fungal spondylodiscitis, particularly caused by *Candida* species. It is essential to promptly recognize this condition, conduct appropriate diagnostic procedures such as imaging studies, and initiate targeted antimicrobial therapy early to enhance patient outcomes and prevent complications. Surgical interventions may be required in situations where there is spinal instability, neurological compression, or advancing infection.
